# Supporting dataset on the content of Cu, Ni Cd, Pb, Zn, Ag, Mg, Fe, Co and Ca in the carcass, gastrointestinal tract tissues and the whole body of nestlings of a small passerine bird, the Eurasian Reed Warbler *Acrocephalus scirpaceus* from an intensively fertilized fishpond habitat

**DOI:** 10.1016/j.dib.2024.111234

**Published:** 2024-12-16

**Authors:** Grzegorz Orłowski, Lucyna Hałupka, Przemysław Pokorny, Bartosz Borczyk, Tomasz Skawiński, Wojciech Dobicki

**Affiliations:** a Institute of Technology and Life Sciences – National Research Institute, Falenty, Al. Hrabska 3, 05-090 Raszyn, Poland; b Ornithological Station, Faculty of Biological Sciences, University of Wroclaw, Sienkiewicza 21, 50-335 Wroclaw, Poland; c Department of Limnology and Fishery, Institute of Animal Breeding, Wrocław University of Environmental and Life Sciences, Chełmońskiego 38C, 51-630 Wrocław, Poland; d Department of Evolutionary Biology and Conservation of Vertebrates, Faculty of Biological Sciences, University of Wroclaw, Sienkiewicza 21, 50-335 Wroclaw, Poland; e Museum of Natural History, Faculty of Biological Sciences, University of Wrocław, Sienkiewicza 21, 50-335 Wrocław, Poland

**Keywords:** Post-natal development, Hatchlings, Elemental composition, Toxic metals

## Abstract

The dataset presented in this data paper supports “The prenatal assimilation of minerals and metals in the nestlings of a small passerine bird” (Orłowski et al. 2024) [1]. The article includes raw data on dead nestlings of a small passerine bird, the Eurasian Reed Warbler *Acrocephalus scirpaceus* breeding in an extensive reedbed (with dominating plant species, the Common Reed *Phragmites australis)* located in an intensively fertilized fishpond habitat, the Stawy Milickie [Milicz Ponds] Nature Reserve (SW Poland). The data include the description of concentrations of Cu, Ni Cd, Pb, Zn, Ag, Mg, Fe, Co and Ca measured in the isolated, emptied gastrointestinal tract, the whole body, and carcass of the each of 26 individual nestlings of a different age (1–9 days old) and hence a different stage of post-natal development. The dataset includes also some additional information on the breeding biology of the focal species.

Specifications Table

**Table:** 

Subject	Ecotoxicology, Ecology, Biological Sciences
Specific subject area	Measurements of concentrations of 10 chemical elements, including two major minerals (Ca and Mg), four essential metals (Co, Cu, Fe, Zn) and four trace metals (Ag, Cd, Ni, Pb) in (1) the isolated, emptied gastrointestinal tract (from the oesophagus to the anus), (2) the whole body, and (3) the body without the gastrointestinal tract (= carcass).
Data format	Raw, analyzed
Type of data	Tables, text file and figures
Data collection	Elemental composition determined using flame atomic absorption spectroscopy (AAS method) on the gastrointestinal tract, the whole body, and the carcass of 26 dead nestlings aged 1–9 days (1d = hatching) from 14 nests collected in 2010–13.
Data source	Raw and analysed.
Data source location	Ornithological Station, Faculty of Biological Sciences, University of Wrocław, Wrocław, Poland.
Data accessibility	Repository name: Zenodo Data identification number: 10.5281/zenodo.13951524 Direct URL to data: https://zenodo.org/records/13951524
Related research article	G. Orłowski, L. Hałupka, P. Pokorny, B. Borczyk, T. Skawiński, W. Dobicki. The prenatal assimilation of minerals and metals in nestlings of a small passerine bird. Science of The Total Environment 954 (2024) 176,437. 10.1016/j.scitotenv.2024.176437

## 1 Value of the Data

• The data present the lengths of (1) three sections of the gastrointestinal tract of Eurasian Reed Warbler *Acrocephalus scirpaceus* nestlings: oesophagus (OE), stomach (ST) and intestine (IN), (2) concentrations of Cu, Ni Cd, Pb, Zn, Ag, Mg, Fe, Co and Ca measured in the isolated, emptied gastrointestinal tract (GT), the whole body (WB), and carcass (CA), and (3) some information on breeding biology, development and biometry of nestlings of the focal species.
• The data in this article should be useful for other researchers to compare the growth of the gastrointestinal tract and tissue accumulation of metals in growing chicks.

## 2 Background

The main source of nutrients for newly hatched nestlings are resources assimilated from eggs, specifically from the yolk sac, until the ingested food becomes the main source of nutrients [2,3]. During nestling development concentrations of elements in different organs change due to metal transfer from ingested food. There is good body of evidence that gastrointestinal tissues constitute the first target for the dietary uptake of chemicals, including toxic trace elements (reviewed by [1]). Gastrointestinal tissues are important in the context of ecotoxicological investigations and environmental assessments of both invertebrates and vertebrates. So far only a few studies have examined changes in the elemental composition of the whole body, the gastrointestinal tract and the contents of gastrointestinal tract in growing nestlings [[4], [5], [6], [7]].

## 3 Data Description

The dataset consists includes the data on sample mass of the carcass and the whole gastrointestinal tract; concentrations of Cu, Ni Cd, Pb, Zn, Ag, Mg, Fe, Co and Ca in the tissues of the gastrointestinal tract, the whole body and the carcass; dates of the laying of the first egg in nests with dead nestlings; age of dead nestlings; distance of nests with dead nestlings from the nearest land, the length of wing, first primary feather, tarsus, oesophagus, stomach and intestine of dead nestlings (Table 1). The differences in concentrations of 10 elements between young nestlings/hatchlings (1–2 d old) and older nestlings (3–9 d old) are presented in Table 2. The results in Table 3 complement the analysis on the potential metal transfer from eggs to hatchlings [1]. To calculate these values we used the elemental concentrations (ppm d.w.) measured in the all previously examined eggs representing embryonated, non-embryonated eggs and eggs with unknown developmental status (*n* = 161 in total) [8], and in the whole body and gastrointestinal tract of hatchlings (1–2 d old; *n* = 5) [1] (Table 4).

**Table 1:** Concentrations of 10 chemical elements (ppm d.w.) measured in three types of samples (carcass, gastrointestinal tract and whole body) of the Eurasian Reed Warbler *Acrocephalus scirpaceus* nestlings sampled in 2010–2013 at the Słoneczny fishpond in the Stawy Milickie Nature Reserve (SW Poland). The study plot represents and extensive reedbed (up to 140 m wide). For each nest the distance between the nest and the nearest land (dike) was given. The whole body concentration is calculated based on the data for carcass and gastrointestinal tract (see [1] for the formula used).

Abbreviations: first primary feather (FPF), oesophagus (OE), stomach (ST) and intestine (IN); * – not determined.

**Table 2:** Results of the Mann-Whitney test showing the differences of 10 elemental concentrations (average±SE) between hatchlings (1–2 d old nestlings) and older nestlings (3–9 d old). The *q*-value is the FDR-adjusted *p*-value (*k* = 10) calculated using the one-stage method. The sample size for hatchlings/older nestlings is 6/20 (carcass), and 5/18 (gastrointestinal tract and whole body).

Element	Hatchlings (1–2 d old)	Older nestlings (3–9 d old)	U	Z	*p*-value	q-value
CARCASS						
Cu	5.41 ± 0.95	8.47 ± 0.73	18.5	−2.53	0.0116	0.0294
Ni	2.35 ± 0.68	0.82 ± 0.10	24.0	2.19	0.0285	0.0356
Cd	1.98 ± 0.33	0.92 ± 0.04	20.0	2.43	0.0149	0.0294
Pb	0.23 ± 0.04	0.11 ± 0.005	20.0	2.43	0.0149	0.0294
Zn	88.12 ± 15.44	71.77 ± 1.69	21.0	2.37	0.0176	0.0294
Ag	0.39 ± 0.11	0.42 ± 0.03	57.0	0.18	0.8551	0.8551
Mg	1040.6 ± 172.3	1982.3 ± 101.1	0.0	−3.65	0.0003	0.0026
Fe	163.6 ± 25.1	292.0 ± 43.1	39.0	−1.28	0.2013	0.2236
Co	0.30 ± 0.05	0.16 ± 0.005	20.0	2.43	0.0149	0.0294
Ca	7272.91750.6	10,854.7 ± 772.6	22.0	−2.31	0.0208	0.0296
GASTROINTESTINAL TRACT						
Cu	17.17 ± 1.48	17.83 ± 1.13	44.0	−0.07	0.9406	0.9406
Ni	4.47 ± 0.59	2.42 ± 0.56	7.0	2.83	0.0046	0.0092
Cd	5.09 ± 0.41	1.53 ± 0.13	0.0	3.35	0.0008	0.0027
Pb	0.43 ± 0.04	0.13 ± 0.01	0.0	3.35	0.0008	0.0027
Zn	84.19 ± 16.24	98.89 ± 5.53	26.0	−1.42	0.1567	0.2239
Ag	0.36 ± 0.09	0.27 ± 0.05	26.0	1.42	0.1567	0.2239
Mg	850.9 ± 224.0	1165.1 ± 74.7	27.0	−1.34	0.1797	0.2247
Fe	265.4 ± 31.2	322.7 ± 46.7	44.0	0.07	0.9406	0.9406
Co	0.88 ± 0.05	0.19 ± 0.02	0.0	3.35	0.0008	0.0027
Ca	18,224.1 ± 4203.8	3124.6 ± 479.7	7.0	2.83	0.0046	0.0092
WHOLE BODY						
Cu	8.99 ± 0.76	10.33 ± 0.69	27.0	−1.34	0.1797	0.2995
Ni	2.90 ± 0.53	1.22 ± 0.22	5.0	2.98	0.0029	0.0057
Cd	2.96 ± 0.27	1.10 ± 0.06	0.0	3.35	0.0008	0.0020
Pb	0.30 ± 0.03	0.12 ± 0.01	0.0	3.35	0.0008	0.0020
Zn	85.31 ± 16.99	79.02 ± 2.59	30.0	1.12	0.2636	0.3295
Ag	0.36 ± 0.11	0.39 ± 0.02	43.5	0.11	0.9110	0.9110
Mg	981.5 ± 159.2	1771.0 ± 94.6	0.0	−3.35	0.0008	0.0020
Fe	192.8 ± 13.3	284.5 ± 33.7	29.0	−1.19	0.2330	0.3295
Co	0.48 ± 0.06	0.17 ± 0.01	0.0	3.35	0.0008	0.0020
Ca	9965.4 ± 1853.5	9237.6 ± 645.0	39.0	0.45	0.6547	0.7275

**Table 3:** Spearman rank correlation coefficients (*r*_s_) between the elemental concentrations (10 elements) in three types of samples (whole body, carcass and gastrointestinal tract), and the length of three sections of the gastrointestinal tract in Eurasian Reed Warbler *Acrocephalus scirpaceus* nestlings; oesophagus (OE; *n* = 22 measurements), stomach (ST; *n* = 23) and intestine (IN; *n* = 12). The statistically significant differences are shown: * – *P* ≤ 0.05, ** – *P* ≤ 0.01, *** – *P* ≤ 0.001.

Section^a^	Cu	Ni	Cd	Pb	Zn	Ag	Mg	Fe	Co	Ca
	WHOLE BODY CONCENTRATION									
OE	0.185	−0.455*	−0.735***	−0.721***	−0.635**	−0.002	0.780***	0.159	−0.697***	0.113
ST	−0.023	−0.485*	−0.494*	−0.512*	−0.122	0.005	0.589**	0.245	−0.404	0.014
IN	0.189	−0.825**	−0.315	−0.112	0.350	−0.014	−0.077	−0.154	−0.608*	−0.077
	CARCASS CONCENTRATION									
OE	0.585**	−0.169	−0.437*	−0.479*	−0.554**	0.107	0.658**	0.219	−0.304	0.634**
ST	0.256	−0.248	−0.393	−0.428*	−0.303	0.066	0.564**	0.262	−0.180	0.515*
IN	0.259	−0.594*	0.119	0.545	−0.077	−0.042	0.028	−0.189	−0.189	0.028
	GASTROINTESTINAL TRACT CONCENTRATION									
OE	0.181	−0.498*	−0.774***	−0.721***	−0.158	−0.059	0.623**	−0.017	−0.682***	−0.388
ST	−0.216	−0.638**	−0.447*	−0.438*	0.368	−0.270	0.351	−0.089	−0.486*	−0.287
IN	−0.203	−0.042	−0.084	−0.105	0.350	−0.147	−0.133	−0.217	−0.273	−0.273

a Notes: (1) Measurements of intestine length were possible in only 12 nestlings, and this was highly skewed towards older nestlings >7d old (*n* = 11); it was measured in only one nestling younger than 4 days (*n* = 13). This problem arises mostly from the small size of the intestine and the high risk of damaging it during preparation. Thus, the results of the relationships between intestine length and elemental concentrations, and nestling age (see below) should be ignored. (2) Spearman's r between the length of each of three sections of the gastrointestinal tract and nestling age for the oesophagus (r_s_ = 0.813, *P* < 0.0001, *n* = 22), stomach (r_s_ = 0.467, *P* = 0.025, *n* = 23) and intestine (r_s_ = 0.111, *P* = 0.111, *n* = 12).

**Table 4:** Potential metal transfer from eggs to nestlings of Reed Warbler expressed as a comparison (average; 95 % CI) of elemental concentrations (ppm d.w.) measured in the contents of eggs representing embryonated eggs, non-embryonated eggs and eggs with unknown developmental status (*n* = 161 in total), and in the whole body and gastrointestinal tract of hatchlings (1–2 d old; *n* = 5). Data on egg elemental concentrations are available elsewhere [8]; both samples, i.e. eggs and nestlings, were collected in 2010–2013. The difference is the quotient of the metal concentration in eggs to concentration in the whole body or gastrointestinal tract; a value of <1 indicates a lower concentration in the eggs.

Element	Eggs^a^	Whole body	Gastrointestinal tract	Difference	
				Eggs/whole body	Eggs/gastrointestinal tract
Cu	16.24	8.99	17.17	1.81×	0.95×
	(13.83–18.64)	(6.87–11.11)	(13.07–21.27)		
Ni	3.25	2.90	4.47	1.12×	0.73×
	(2.76–3.74)	(1.42–4.39)	(2.84–6.09)		
Cd	1.22	2.96	5.08	0.41×	0.24×
	(1.05–1.38)	(2.21–3.72)	(3.96–6.23)		
Pb	5.91	0.30	0.43	19.7×	13.74×
	(5.00–6.82)	(0.21–0.39)	(0.32–0.54)		
Zn	91.6	85.3	84.2	1.07×	1.09×
	(78.1–105.2)	(38.1–132.5)	(39.1–129.3)		
Fe	184.5	192.8	265.4	0.96×	0.70×
	(161.8–207.1)	(155.8–229.8)	(178.8–352.0)		
Co	5.63	0.48	0.87	11.7×	6.47×
	(5.04–6.23)	(0.32–0.65)	(0.72–1.03)		
Mg	679.8	981.5	850.9	0.69×	0.80×
	(615.7–743.9)	(539.4–1423.6)	(229.9–1472.7)		
Ca	11,582	9965	18,224	1.16×	0.64×
	(10,247–12,918)	(4819–15,112)	(6552–29,896)		

a The dry weight for elemental concentrations in egg contents was estimated from the wet weight (determined for raw, unprocessed eggs; *n* = 161) based on the results of our previous study on the same population of Reed Warbler [8]; concentrations from this output data were multiplied by 5.05; this conversion was based on the average moisture content (80.16 %) of eggs calculated from data given by Mora (2003) [14] for 11 species of small passerines; similar conversion factor (×5) was applied by Jopek et al. 1995 [15].

## 4 Experimental Design, Materials and Methods

The data were collected in 2010–2013 on a 3 ha study plot at Słoneczny fishpond in the Stawy Milickie [Milicz Ponds] Nature Reserve (SW Poland), where we have been studying the population of Eurasian Reed Warblers since 2006 [[8], [9], [10]]. Throughout the breeding seasons (May–August) we searched for nests of the species, individually marked parents (metal and colour rings) and identified them at the nests. We visited nests usually every second day, but daily before the expected hatching day in order to accurately determine the hatching date and the age of the young. If a nest with nestlings was found, the nestling age was estimated using data from [11].

**Fig. 1:**
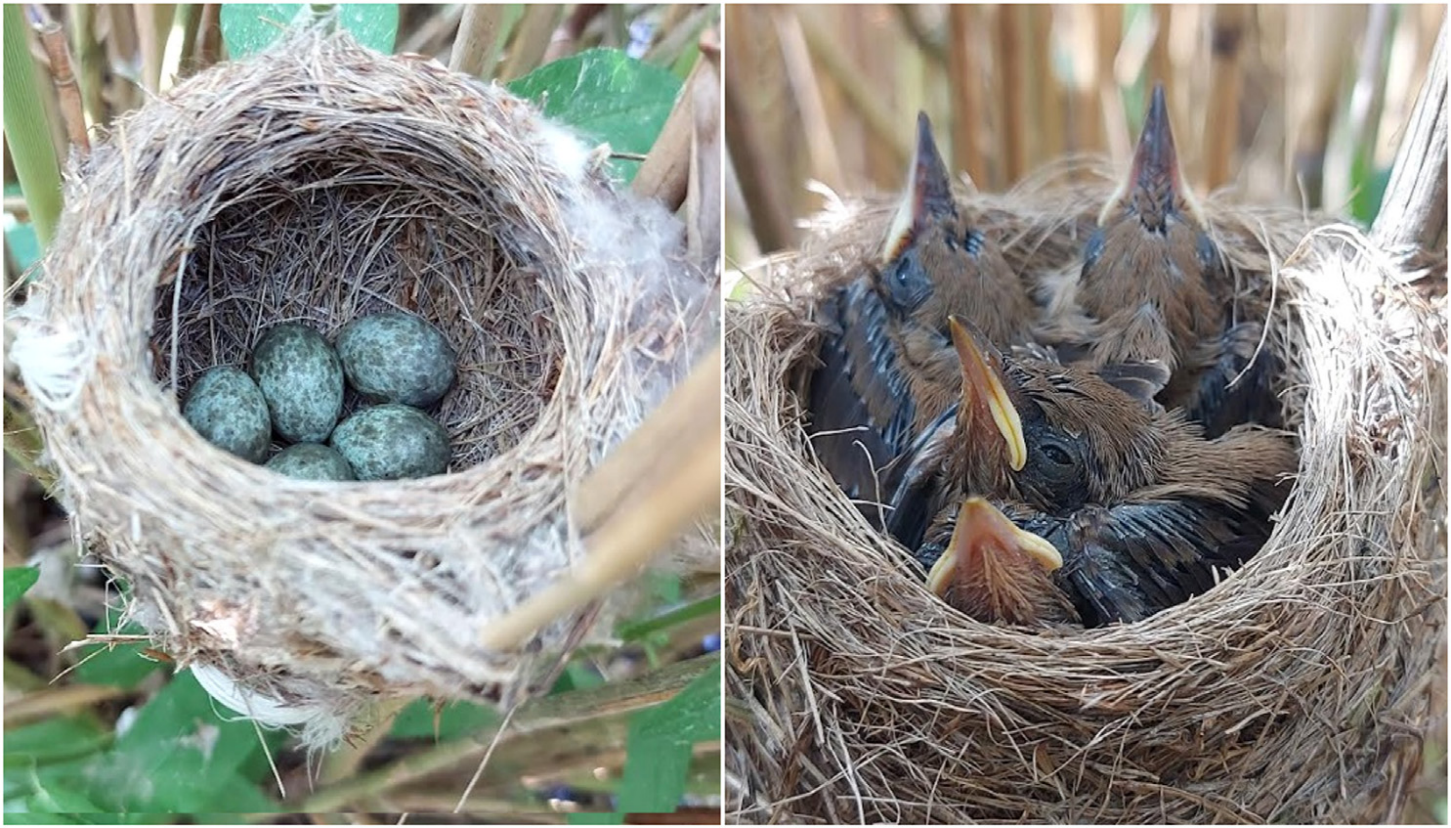
The five-egg clutch (left) and 9-day-old nestlings of the Eurasian Reed Warbler *Acrocephalus scirpaceus* on the Słoneczny fishpond in the Stawy Milickie Nature Reserve (SW Poland).

During four breeding seasons we found and collected 26 dead nestlings aged 1–9 days (1d = hatching) from 14 nests (Fig. 1, Table 1). On the day of collection we placed the nestlings' bodies in a freezer (-20 °C). In 2018 we removed the gastrointestinal tracts from the nestling bodies and emptied them. We also measured the lengths of the oesophagus, stomach and intestine of each nestling to the nearest millimetre using an ornithological ruler, if only it was possible (Table 1). Each measurement was repeated three times, and mean values were calculated and used in all analyses.

Prior to the elemental analysis all samples were stored at 105 °C to obtain their dry body mass; for more details see [1]. After drying, the samples (bodies without digestive systems and gastrointestinal tracts separately) were weighed to the nearest 0.1 mg. Afterwards the samples were homogenized by grinding them with a porcelain pestle and mortar. The next step was wet mineralization with nitric acid (HNO_3_) in a MARS 5 (CEM, USA) pressure microwave oven. The content of 10 elements (Ag, Ca, Cd, Co, Cu, Fe, Mg, Ni, Pb, Zn) in the mineralizate was estimated using flame atomic absorption spectroscopy (SpectrAA FS220; Varian, Australia). Element concentrations were expressed in milligrams per kilogram (mg kg^−1^ or parts per million; ppm) of dry sample weight (d.w.). From the concentrations and dry weights of the whole samples, the concentrations of the elements in the whole bird body were calculated using the formula provided in our main article [1].

We used the Mann-Whitney test to assess the differences in concentrations 10 elemental concentrations between hatchlings (1–2 d old nestlings) and older nestlings (3–9 d old) (Table 2). Table 2 summarizes the original *p*-values from these tests. However, due to the large number of paired comparisons, we applied False Discovery Rate (FDR) procedure to adjust the original *p*-values using the classical one-stage method [12] in the statistical software spreadsheet [13]. The FDR-adjusted *p*-values were calculated from 10 elemental concentration tests (*k* = 10).

## Limitations

Not applicable.

## Ethics Statement

The authors have read and followed the ethical requirements for publication in Data in Brief, and confirm that the current work does not involve human subjects, animal experiments, or any data collected from social media platforms. The permits to conduct the study were issued by the Lower Silesian Regional Directorate of Nature Protection in Wrocław, Poland (RDOŚ-02-WPN-6630/69/10/mr; WPN.6205.55/2011.MR; WPN.6205.39/2012.MR; WPN.6205.38.2013.MR2).

## CRediT Author Statement

All authors discussed the results and contributed to its final version. All authors wrote the manuscript. Grzegorz Orłowski and Lucyna Hałupka conceived the idea of this investigation and supervised the progress in the study, data collection and analyses. Lucyna Hałupka with her research team collected and measured the nestlings, and prepared the photographic documentation. Bartosz Borczyk and Tomasz Skawiński prepared the tissue samples for chemical analysis. Przemysław Pokorny and Wojciech Dobicki conducted the chemical analysis of samples. Grzegorz Orłowski and Lucyna Hałupka conducted the data treatment.

## Data Availability

zenodoDataset on the content of Cu, Ni Cd, Pb, Zn, Ag, Mg, Fe, Co and Ca in the carcass, gastrointestinal tract tissues and the whole body of nestlings of a small passerine bird, the Eurasian Reed Warbler A (Original data).
